# A Systems Biology Approach to Uncovering Pharmacological Synergy in Herbal Medicines with Applications to Cardiovascular Disease

**DOI:** 10.1155/2012/519031

**Published:** 2012-11-29

**Authors:** Xia Wang, Xue Xu, Weiyang Tao, Yan Li, Yonghua Wang, Ling Yang

**Affiliations:** ^1^Center of Bioinformatics, College of Life Science, Northwest A&F University, Yangling, Shaanxi 712100, China; ^2^School of Chemical Engineering, Dalian University of Technology, Dalian, Liaoning 116024, China; ^3^Institute of Chinese Materia Medica, China Academy of Chinese Medical Sciences, Beijing 100700, China; ^4^Laboratory of Pharmaceutical Resource Discovery, Dalian Institute of Chemical Physics, Chinese Academy of Sciences, Dalian 116023, China

## Abstract

*Background*. Clinical trials reveal that multiherb prescriptions of herbal medicine often exhibit pharmacological and therapeutic superiority in comparison to isolated single constituents. However, the synergistic mechanisms underlying this remain elusive. To address this question, a novel systems biology model integrating oral bioavailability and drug-likeness screening, target identification, and network pharmacology method has been constructed and applied to four clinically widely used herbs Radix Astragali Mongolici, Radix Puerariae Lobatae, Radix Ophiopogonis Japonici, and Radix Salviae Miltiorrhiza which exert synergistic effects of combined treatment of cardiovascular disease (CVD). *Results*. The results show that the structural properties of molecules in four herbs have substantial differences, and each herb can interact with significant target proteins related to CVD. Moreover, the bioactive ingredients from different herbs potentially act on the same molecular target (multiple-drug-one-target) and/or the functionally diverse targets but with potentially clinically relevant associations (multiple-drug-multiple-target-one-disease). From a molecular/systematic level, this explains why the herbs within a concoction could mutually enhance pharmacological synergy on a disease. *Conclusions*. The present work provides a new strategy not only for the understanding of pharmacological synergy in herbal medicine, but also for the rational discovery of potent drug/herb combinations that are individually subtherapeutic.

## 1. Introduction

Herbal medicine, especially traditional Chinese medicine (TCM) with the longest history in Asia, is a cost-effective system of medical practice that differs in substance, methodology, and philosophy to modern medicine, and plays an important role in health maintenance for the peoples of the world [1]. Because of their extensive use and the therapeutic effects [2, 3], there is an increasing interest and need to evaluate the mechanisms of action of herbal products rigorously.

Herbal medicines are characterized by the use of mixtures of several herbs (multiherbs) in a single formula, in which the pharmacological activities of one single herb is either potentiated or prolonged, and/or its adverse effects reduced by addition of other herbs [4]. This thus will lead to a more favorable response for some herbal combinations than for the constituent herb used alone [2], which suggests that therapeutic effects of these herbal products may arise from synergistic actions of herbal ingredients [3, 4].

Up to now, herbal synergism has been frequently reported [5], which may result from: (1) the potentiation of pharmacokinetics, such that one ingredient enhances the therapeutic effect of another component by regulating the drug absorption and metabolism. For examples, saponins increase absorption of corticosteroids [6] and procyanidin B2 or hyperoside in St. John's wort increases the solubility of hypericin [7, 8]; and (2) reinforcement of pharmacodynamics, thus all ingredients involved in an herbal combination direct at a similar receptor target or physiological system [4]. For instance, in the case of St. John's wort, individually subtherapeutic effects (e.g., MAO and COMT inhibition) may combine to augment the primary pharmacological mechanism (monoamine reuptake inhibition). However, the molecular mechanism underlying such multicomponent synergy associated with the interacting targets, pathways, and even diseases remains largely uncovered. Clearly, deep understanding of the herb synergism will be not only helpful to optimize the drug combinations in multicomponent therapeutics but also critical for developing novel drug combinations that are individually subtherapeutic but efficacious in combination.

Evaluation of synergy in multicomponent therapeutics is usually performed experimentally in a case-by-case approach [9]. For examples, Wiesner et al. demonstrated that a novel antimalarial drug fosmidomycin had both *in vitro* and *in vivo* synergic effect with clindamycin [10]; Nguyen et al. reported that triple combination of amantadine, ribavirin, and oseltamivir was highly active and synergistic against drug resistant influenza virus strains *in vitro* [11]. However, as we know for any medicinal herb, they might contain hundreds of ingredients, thus it is unfeasible to screen all possible drug combinations for all possible indications, although high-throughput screening was possible to determine drug combinations [12], it is also much expensive. Another drawback of the existing methods is that these “blind” approaches including molecular biology are costly and time consuming. In addition, little is known about the system properties of a full drug interaction network, which hinders the understanding of mechanisms of drug combinations.

Alternatively, computational methods, especially systems biology, that enable to investigate the complex mechanism of action of drugs and circumvent the challenges associated with experiment have recently been developed. Systems biology investigates the biological processes within the complex, physiological milieu systematically through a systems approach integrating experimental, mathematical, and computational sciences. It has the potential to further facilitate the identification and validation of the therapeutic modulation of regulatory and metabolic networks and hence help identify targets and biomarkers, as well as ‘‘off-target” and side effects of drug candidates (reviewed by [13]). For example, network-target based techniques were used for virtual screening synergistic drug combinations [14, 15], thereby try to explain how and why the drugs work. But they only work on small drug sets due to the computational and experimental cost. Moreover, drugs are generally combined based on their mechanisms of action, which is characterized by the properties of drugs, such as their targets and pharmacology [16]. Thus the incompleteness of molecular networks and the scarceness of the drug properties limit the application of such approaches to TCM considerably.

In this work, we present a novel concept based on a systems biology framework for the investigation of synergy of four herbs, that is, Radix Salviae Miltiorrhiza (RSM), Radix Astragali Mongolici (RAM), Radix Puerariae lobatae (RPL), and Radix Ophiopogonis Japonici (ROJ) [17]. Among these four herbs, RSM shows diverse biological activities, such as inhibition of angiotensin converting enzyme (ACE), lowering blood pressure, dilate arteries, and decreasing blood clotting [18–20], thus is widely prescribed in different TCM formulae; RAM also shows protective effects against ultraviolet A-induced photoaging in human fibroblasts [21] and on proliferation and Akt phosphorylation of breast cancer cell lines [22]; isoflavones from RPL and their metabolites can inhibit growth and induce apoptosis in breast cancer cells [23]; ROJ plays a role in enhancing immunity, anti-myocardial ischemia, lowering blood glucose, and antiviral activity [24]. Impressively, RSM has shown synergisms with each of the other three herbs in clinical trials for cardiovascular disease (CVD) [17], the leading cause of morbidity and mortality all over the world [25].

## 2. Materials and Methods

We have identified the potential targets of the four herbs on a proteome-wide scale and disclosed the synergistic mechanisms of action of the active ingredients by integrating both molecular and pharmacological features associated with drugs [26, 27]. Our methodology effectively and systematically extends the scope of the previously network-target concept, and is more likely to be successful in achieving the ultimate goal of providing pharmacological synergy in psychoactive herbal medicines. Our systems biology approach proceeds as follows:

1. all 3D structures of available molecules in the four herbs are collected;
2. the drug-likeness (DL) and oral bioavailability (OB) of the molecules are calculated to prescreen for the bioactive molecules;
3. the physicochemical properties and architecture of molecules in four herbs are revisited;
4. the potential targets of these four herbs are identified on a proteome-wide scale;
5. the tools of network biology and systematic information about drugs and their targets are combined to uncover the synergistic therapeutic actions of herbal ingredients.

Our approach essentially explores some feature patterns enriched in known combinatorial therapies that can be predictive of new drug combinations and provide insights into the mechanisms underlying combinatorial therapy. Then these compounds and proteins are mapped to functional ontologies such as compound-target associations, compound-pathway connections, and disease-target; we assessed retrospectively and prospectively network-based relationships between drugs and their targets and interrelationships between drug targets and disease-gene products. In this way, our approach has the potential to increase the rate of successful drug discovery and development.

### 2.1. Database Construction and Molecular Modeling

In order to extract the current state of the art on known chemical ingredients in these four herbs, their information was extracted from the Traditional Chinese Medicine Systems Pharmacology database and Analysis Platform (TcmSP; http://tcmspnw.com/) that was recently developed in our group. The newest version of TcmSP comprises 510 effective herbal entries registered in Chinese pharmacopoeia with more than 31000 ingredients, which spread over 18 different drug classes. This is currently the most comprehensive small molecular database for systems pharmacology analysis of TCM. In this work, the 3D structures of all molecules in the database were minimized in Sybyl by using the standard Tripos force field (Tripos, Inc.). After removing the duplicated compounds, a total of 532 molecules with 209 in RSM, 95 in RAM, 113 in RPL, and 135 in ROJ were collected in this study. Glycosides in medicinal herbs are usually hydrolyzed to liberate aglycone which is then absorbed at the intestinal mucos [28], thus the corresponding aglycone chemicals were also added into the database.

### 2.2. Drug-Likeness Calculation

DL is a qualitative concept used in drug design for how ‘‘druglike” a substance is with respect to factors affecting pharmacodynamics and pharmacokinetics of molecules which ultimately influences their absorption, distribution, metabolism, and excretion (ADME) in human body [29]. DL between the compound structure *x* and the drug molecular structure *x*′ obtained from Drugbank was evaluated by Tanimoto coefficient defined as:

(1) $$\begin{array}{c} f\left(A,B\right)=\frac{A*B}{{\left|A\right|}^{2}+{\left|B\right|}^{2}-A*B}, \end{array}$$

in which *A* represents the new compound, and *B* represents the average drug-likeness index of all 6511 molecules in DrugBank database (access time: June 1st, 2011, http://www.drugbank.ca/). In this work, compounds with DL ≥ 0.2 were selected as the candidate bioactive molecules, because the mean value of DL for all 6511 molecules in DrugBank is 0.18.

### 2.3. Oral Bioavailability Prediction

Herbal medicine is administrated mainly by oral route. In the development of herbal drugs intended for oral use, good drug absorption and appropriate drug delivery are very important. OB, the percentage of an oral dose able to produce a pharmacological activity, is one of the most desirable attributes of a new drug. To calculate the OB, we have developed a robust in-house system OBioavail 1.1 [27] integrating the metabolism (cytochrome P450 3A4) and transport (P-glycoprotein) information. This program helped us screen out the compounds with good OB, thus significantly reducing the number of original components to a smaller set for herbal medicine.

In this work, compounds with OB ≥ 40% were selected as the candidate bioactive molecules. The threshold determination is based upon careful consideration of the following: (1) information from the studied herbs is extracted as much as possible using the least number of chemical ingredients; (2) the obtained model can be reasonably explained by the reported pharmacological data.

### 2.4. Comparisons of Four Herbs Based on Chemicals

To analyze differences in molecular properties and structural features between RSM and the other three herbs, eight representative drug-related physicochemical properties including molecular weight (MW), number of rings per molecule (nCIC), octanol-water partition coefficient (MlogP), hydrogen bond donors/acceptors (nHDon and nHAcc), number of rotatable bonds (RBN), hydrophilic factor (Hy), and topological polar surface area (TPSA) were calculated with the dragon software [30]. To obtain a visual representation of the property space, property distribution analyses of both total compounds and bioactive chemicals were carried out considering all eight of the above mentioned physicochemical properties.

### 2.5. Target Identification

In silico prediction of drug-target interactions from heterogeneous biological data can accelerate the system-level search for drug molecules and therapeutic targets. Recently, we have developed a robust model [26] that efficiently integrates chemical, genomic, and pharmacological information for drug targeting and discovery on a large scale, based on random forest (RF) and support vector machine (SVM) methods. The optimal models show impressive reliability of prediction for drug-target interactions, with the concordance of 85.83%, the sensitivity of 79.62%, and the specificity of 92.76% [26]. In this work, the predicted targets for each bioactive molecule were selected based on the following principles [26, 31]: firstly, the targets should be both presented in the RF and SVM positive prediction list (value > 0.5); secondly, the targets with value of greater than 0.7 for RF and 0.8 for SVM were chosen as the final predicted targets. At last, a total of 87 targets were reserved for further analysis.

### 2.6. Network Construction

The predicted targets were used to build the compound-target networks by linking with the bioactive compounds. The relationship between CVD and targets was retrieved from the PharmGkb database [32] (http://www.pharmgkb.org/) and therapeutic target database [33] (http://bidd.nus.edu.sg/group/ttd/ttd.asp). In the interactive network diagram, nodes represent either compounds or proteins, and edges indicate compound-target interactions. All networks were generated in Cytoscape 2.8.1, a popular bioinformatics package for biological network visualization and data integration [34]. The quantitative properties of these networks were analyzed by two plugins including NetworkAnalyzer and CentiScaPe 1.2.

## 3. Results and Discussion

For thousands of years, herbal medicine holds a great promise for medical diagnosis and treatments in Asia and now is considered a complementary or alternative medical system in most Western countries. Different from conventional medicine in which drugs are studied in isolation, herbal medicine typically incorporates several medicinal herbs which contain multiple ingredients that probably produce a more favorable response than an isolated single constituent [4]. Such multicomponent therapeutics thereby is considered as a rational and efficient form of therapy designed to control complex diseases such as CVD [35]. However, despite the fact that many positive outcomes have been observed in *in vivo* studies [36] and clinical trials [3], the underlying mechanisms of action, especially the synergism, remain to be elucidated for many psychoactive herbal medications. In this study, we applied our method to three representative herb pairs, that is, RSM and RAM, RSM and RPL, RSM and ROJ, with applications to CVD.

### 3.1. Extracting Active Components by OB and DL Prescreening

The oral route of drug administration is the most convenient way of choice for the formulators and continues to dominate the area of TCM therapy. However, though popular, this route is limited by absorption and bioavailability in the milieu of gastrointestinal tract. Hence OB is undoubtedly one of the most important pharmacokinetic parameters since it is the indicator of the efficiency of the drug delivery to the systemic circulation. Furthermore, a drug-like compound often has sufficiently acceptable ADME properties and can exert the pharmacodynamic effect on target site in human body. In short, the OB and DL prescreening is favorable to determine pharmaceutically active compounds in medicinal herbs.

#### 3.1.1. RSM

A total of 40 compounds with good OB (≥40%) and DL value (≥0.2) are obtained (Table 1), most of which have been reported as bioactive ingredients. For examples, miltirone II (OB = 45%, DL = 0.24) presents sedative activity and is a benzodiazepine receptor agonist [37]; cryptotanshinone (OB = 52%, DL = 0.46) and tanshinone VI (OB = 53%, DL = 0.36) can protect the myocardium against ischemia-induced derangements by eliciting a significant enhanced recovery of the contractile force upon reoxygenation [38]. This further validates the reasonability of our prescreening model. According to the prescreening rule, we also find some other potential bioactive compounds including tanshinaldehyde and tanshinone VI that have not yet been validated. Notably, although salvianic acid A and Tanshinone II B have low DL or OB (0.06 and 22%, resp.), both of them exhibit significant biological activities. For instances, salvianic acid A exerts protective effect on vascular endothelial cells induced by lipopolysaccharide via an antioxidative mechanism, thus inhibiting apoptotic morphological changes of cells [39]; Tanshinone II B (DL = 0.45) exerts neuroprotective effect via inhibition of neuronal apoptosis *in vitro* [40]. Therefore, these two compounds are also involved as the bioactive ingredients of RSM. In summary, 42 (20.6% of all 209) bioactive compounds from RSM are reserved for further target prediction.

#### 3.1.2. RAM

In this herb, total 44 (46.3% of all 95) compounds (Table 1) are obtained after prescreening of OB and DL and reserved for further target prediction. Most of them belong to astragalus flavonoids which have shown well protective effects on CVD [41]. For examples, calycosin (OB = 89%, DL = 0.24) and kaempferol (OB = 66%, DL = 0.24) are demonstrated to have antioxidant activities that benefit for CVD [42]; aglycon of formononetin-7-glucoside (formononetin, OB = 62%, DL = 0.21) reduces circulating concentrations of VCAM-1 which is an adhesion molecule associated with atherosclerosis [43]; quercetin (OB = 13%, DL = 0.27) may exert multiple actions on the NO-guanylyl cyclase pathway, endothelium-derived hyperpolarizing factor(s) and endothelin-1 and protect endothelial cells against apoptosis [41], thus epidemiologically associated with protection from coronary artery disease and cancer [44]. Besides, some molecules belong to triterpenoid saponins which are responsible for the bioactivities and efficacies of RAM on the treatment of CVDs. For instance, after deglycosylation, astragalosides I–IV with high OBs (range from 41% to 81%) can prevent changes of sarcoplasmic reticulum Ca^2+^-uptake ability and Ca^2+^-ATPase and Ser16-phosphorylated phospholamban protein expression, thus may prevent the depression in sarcoplasmic reticulum Ca^2+^ transport and improve cardiac function [45]. In addition, gamma-aminobutyric acid (GABA) with a high OB of 62% but low DL value (0.01) is also analyzed since it is reported to be the main hypotensive principle of right anterior measurement [46].

#### 3.1.3. RPL

A total of 12 (10.6% of all 113) potential bioactive compounds (Table 1) are obtained for further target prediction. Most of them come from pueraria isoflavones, which are the main active constituents of RPL [47]. Notably, although daidzin has been demonstrated as a potent, selective inhibitor of human mitochondrial aldehyde dehydrogenase [48], its OB is extremely low (10%). Interestingly, its aglycon (daidzein) possesses relative good OB (38%) and affords protection against CVD [49]. Thus we propose that daidzin serves as a prodrug that is metabolized to the active form by intestinal bacterial deglycosylation. Similarly, after deglycosylation, the compound ononin (formononetin 7-O-*β*-D-glucoside) has good OB and presents certain pharmacological effects. Although puerarin in RPL has low OB (13%), it is the most abundant isoflavone glucoside and found to act as a *β*-adrenoreceptor antagonist in isolated arteries and veins [50]. Thus this molecule is also involved as active ingredients.

#### 3.1.4. ROJ

In this herb, 14 compounds with satisfied drug property (OB > 40%; DL > 0.2) are obtained. Most of them belong to homoisoflavonoids which have one more carbon atom than normal isoflavone skeleton. For example, ophiopogonanone B, G, ophiopogonone A, 6-aldehydoisoophiopogonone A, methylophiopogonanone A and B are responsible for bioactivities including antioxidant activity, inhibition of platelet aggregation, cough relief, and hyperglycemia [51–53]. In addition, DL prediction of ophiopogonin D gives a relatively low value of 0.08, but it is also included for further analysis due to its various biological activities, such as inhibition of venous thrombosis, anti-inflammation, and antitussive activity [54]. Similarly, ophiopogonone A, 6-aldehydoisoophiopogonone A, methylophiopogonanone A, and methylophiopogonanone B displaying significant biological activities are also included though have low OBs (from 5% to 19%). To sum up, 19 (14.1% of all 135) candidate compounds are used for further target prediction (Table 1).

### 3.2. Potential Target Identification

To develop effective and safe therapies is the ultimate goal of medicine. For TCM comprising many molecules, a key challenge remains the identification of the molecular targets underlying the beneficial or detrimental effects of drugs. Thus a systematic, widely applicable, and robust approach is badly needed. Previously, we have developed a simple, universally applicable target identification approach on the basis of the RF and SVM techniques. In this section, with application of this method, we have analyzed the binding of Chinese herbs RSM, RAM, RPL, and ROJ to targets of interest in the CVD.

#### 3.2.1. RSM

There are 68 potential targets identified for 43 bioactive components in RSM (supporting Table S1). This means that one compound hits 1.6 target proteins on average, which elaborates the polypharmacology characteristic of the multicomponent TCM. Among 68 targets, 21 are proven to be related with CVD. For examples, 5-hydroxytryptamine 2A receptor, alpha-1D adrenergic receptor, and beta-2 adrenergic receptor are reported to play an important role in the pathogenesis of hypertension [55–57]; potassium voltage-gated channel and sodium channel protein are demonstrated to be related with cardiac arrhythmias [58, 59]; prothrombin is proved to have a relationship with myocardial infarction, stroke, and venous thrombosis in a large cohort of US men [60].

#### 3.2.2. RAM

A total of 77 target proteins (supporting Table S1 in Supplementary Materials available online at doi:10.1155/2012/519031) are obtained for 44 bioactive compounds in this herb, of which 19 are CVD related. For examples, peroxisome proliferator-activated receptor gamma (PPAR*γ*) is expressed by macrophages, endothelial cells, and vascular smooth muscle cells. It regulates gene expression of key proteins involved in lipid metabolism, vascular inflammation, and proliferation contributing to atherogenesis and postangioplasty restenosis, thus having beneficial effects on CVD [61]; vascular endothelial growth factor receptor 2 can regulate angiogenesis which is a critical reparative process that occurs subsequent to ischemic injury [62].

#### 3.2.3. RPL

In this herb, 12 candidate drugs are predicted to bind with 34 target proteins (supporting Table S1), of which 13 link with CVD. For example, the major active constituent puerarin (M64) hits 3 potential targets associated with CVD such as estrogen receptor, prostaglandin G/H synthase 2, and prothrombin. Recently, puerarin has been reported to compete with 17*β*-estradiol binding to estrogen receptors, thereby suppressing invasion and vascularization of endometriosis tissue stimulated by 17*β*-estradiol [63]. Besides, puerarin modulating the proteins prostaglandin G/H synthase 2 and prothrombin gives insights into the puerarin-induced protection against myocardial infarction and improvement of the blood flow [64]. These findings may explain why puerarin is responsible for the pharmacological effects of RPL on the cardiovascular systems in several animal models with cardiovascular disorders [65].

#### 3.2.4. ROJ

Altogether 77 target proteins (supporting Table S1) identified for 19 candidate drugs in this herb. Among 77 targets, 19 are relevant to CVD such as protein beta-2 adrenergic receptor related with disease myocardial ischemia; potassium voltage-gated channel and sodium channel protein associated with arrhythmia; 5-hydroxytryptamine 2A receptor related with thrombosis, which clarifies the herbal cardiovascular activities such as anti-ischemia, anti-arrhythmic, and antithrombotic. Impressively, multiple targets of this herb such as sodium channel protein, beta-2 adrenergic receptor, and 5-hydroxytryptamine 2A receptor, are shared with RSM.

### 3.3. Differences in Chemical Space of the Four Medicinal Herbs

**Figure 1:**
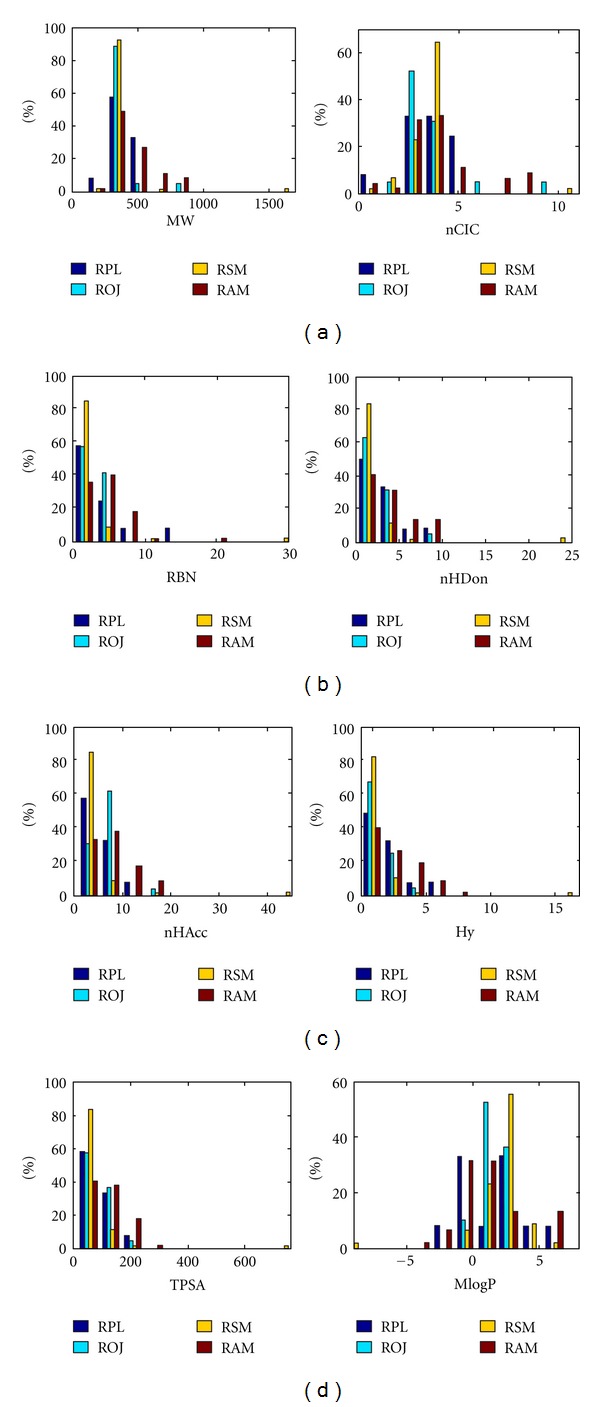
The profile distributions of eight important molecular properties for bioactive molecules from Radix Salviae Miltiorrhiza (RSM), Radix Astragali Mongolici (RAM), Radix Puerariae lobatae (RPL), and Radix Ophiopogonis Japonici (ROJ).

In TCM, pharmacological activities of one herb are usually potentiated by addition of other herbs in a formula. The nature of such pharmacological synergy in psychoactive herbal medicine is probably due to the bioactive compounds targeting a similar receptor or physiological system [4]. Since the chemical composition of herbs will provide the building blocks of the pharmacology activities, a question arises whether herbs of similar pharmacology activity (treatment of CVD) have similar chemical composition. To answer this question, we have analyzed the molecular diversity of compounds from four herbs by considering 8 common descriptors including MW, nCIC, RBN, nHDon, nHAcc, Hy, TPSA, and MlogP for these four compound classes (Table 2, Figure 1 and supporting Figure S1 available online at doi:10.1155/2012/519031), since these parameters can reflect the basic characteristics of a molecule especially its pharmacodynamic properties.

Generally, systematic investigations of chemical space are used as a way of measuring the diversity of a compound library. The main focus of this study is on comparing scaffolds of bioactive natural products inherent to different medicinal herbs, thus for further understanding of scaffold architectures in different herbs that might be suitable for combinatorial library design. We get a first overview of molecular distribution of all molecules in each herb. The distribution curves of different properties are displayed in supporting Figure S1 and the mean values are in Table 2. The distribution characteristics of each descriptor for all herbs are similar to those of natural products observed by Feher and Schmidt [66]. Although the distribution of each descriptor for compounds in RSM peaks at a similar position as those in the other three herbs, they are skewed toward much lower values except MlogP. Besides, RSM has an obviously narrower distribution of each descriptor than that of the other herbs, suggesting that compounds in RSM are substantially less diverse than those in the other three herbs. These results demonstrate that chemical compositions of RSM and the other three herbs have substantially different properties. The following section will be devoted to comparison of molecular distribution of bioactive molecules in each herb.

As shown in Table 2, the average calculated MW is similar for ROJ (373.42 ± 120.83) and RPL (379.77 ± 92.05), which is much lower than that of molecules in RAM (438.31 ± 169.88) while significantly higher than those of RSM (343.28 ± 219.76); the peaks of MW distribution of four (bioactive) natural products' datasets are located at around 300~500. As shown in Figure 1, the MW distribution of RSM is highly overlapped with those of the other three herbs and slightly skewed toward lower molecular weights.

The average lipophilicity MlogP value is the highest for the RSM (2.10 ± 2.28), followed by ROJ (1.61 ± 0.91), RPL (1.44 ± 2.57), and RAM (1.18 ± 2.60) (Table 2 and Figure 1), indicating that the molecules in RSM are more soluble in neutral solvents than those from the other herbs. From Figure 1, interestingly, we find that RSM has a wider distribution of MLogP than ROJ but less than RAM and RPL. Similarly, the mean value of hydrophilic factor for each molecule is the lowest for RSM (0.38 ± 2.79), followed by ROJ (0.74 ± 0.97), RPL (1.28 ± 1.61), and RAM (1.68 ± 1.95). This result indicates that molecules in RSM are much more hydrophobic than molecules in the other herbs.

The average number of rotatable bonds (RBN) per molecule is the lowest for RSM (2.09 ± 4.99), followed by ROJ (3.26 ± 1.45), RPL (4.08 ± 3.96), and RAM (5.02 ± 3.42). Considering the mean MW of ROJ that is almost equal to that of the RPL, chemicals in RPL are probably more flexible than those in ROJ. Notably, when two molecules with different flexibility follow the same interaction with a target, the rigid molecule has a relative lower entropy loss compared with the flexible one, thus usually leading to the stronger binding affinity of rigid molecule [66]. This finding suggests that the flexibility of a molecule plays a key role in determining its binding to the active site of a target. Therefore, molecules in RPL probably have more thermodynamic advantages to achieve favorable binding properties than those in the ROJ. The prevalence of rings is another measurement for the rigidity of molecules, similar to RBN, the average number of rings per molecule (nCIC) in RAM (4.14 ± 1.84) is the lowest among four classes.

For nHDon and nHAcc, RSM has the least donor/acceptor atoms for H-bonds (HDon = 1.95; nHAcc = 5.19), while the RAM (HDon = 3.89; nHAcc = 7.91) has the largest number, followed by RPL (HDon = 3.42; nHAcc = 6.42) and ROJ (HDon = 2.68; nHAcc = 6.58) has mediate ones. An examination of the frequency of occurrence of elemental composition reveals that molecules in all herbs have many oxygen atoms but few nitrogen atoms. On average, the total number of oxygen and nitrogen atoms for RSM, RAM, ROJ, and RPL are 5.19, 7.64, 6.53, and 6.42, respectively. Therefore, among four herbs, compounds in RAM have the most polar functional groups which can act as H-bond acceptors.

TPSA is a key factor for OB and typically compounds are considered to be orally bioavailable with a TPSA value between about 80 and 150 Å^2^ [67]. The peaks of TPSA distribution of four herbs locating around this range further validate our prescreening model. RAM (123.18 ± 69.35 Å^2^) has the highest average TPSA values, followed by and RPL (106.28 ± 59.74 Å^2^), ROJ (100.13 ± 35.49 Å^2^), and RSM (86.86 ± 114.01 Å^2^). This is similar to the trends of nHDon and nHAcc since TPSA is relevant to the number of hydrogen-bond donors and acceptors.

In summary, the distribution profiles of these basic, physicochemical properties for RSM are obviously different from those of the other three herbs, suggesting four herbs with different chemical compositions. However, their pharmacological roles are very similar in treatment of CVD. It is reasonable to speculate that their real interaction with target proteins may be different. Therefore, the mutual enhancement of these herb pairs could be achieved through the different mode of actions to exert a complementary synergistic effect (details in the next section).

### 3.4. Uncovering the Synergy from Network Pharmacology Level

Herbal medicines are multicomponent therapeutics, in which two or more herbs interact with multiple targets simultaneously, thus are considered as a rational and efficient form of therapy designed to control complex diseases [35]. The fundamental advantage of this therapeutics is the generation of synergism between ingredients (i.e., herbs and/or phytochemicals) within herbal formulas when two or more ingredients within a concoction mutually enhance the effect of the formulation in a certain activity or clinical outcome [68]. A representative herb RSM was reported to have synergic effect with multiple different herbs including RAM, RPL, and ROJ, and clinical trials have demonstrated that multiherb RSM formula enhanced stroke survival and recovery in comparison to RSM alone [2]. However, the molecular mechanism underlying the herbal synergism remains unclear. To solve this problem, the network pharmacology has been employed to understand the multicomponent synergy by its latent network topology properties.

**Figure 2:**
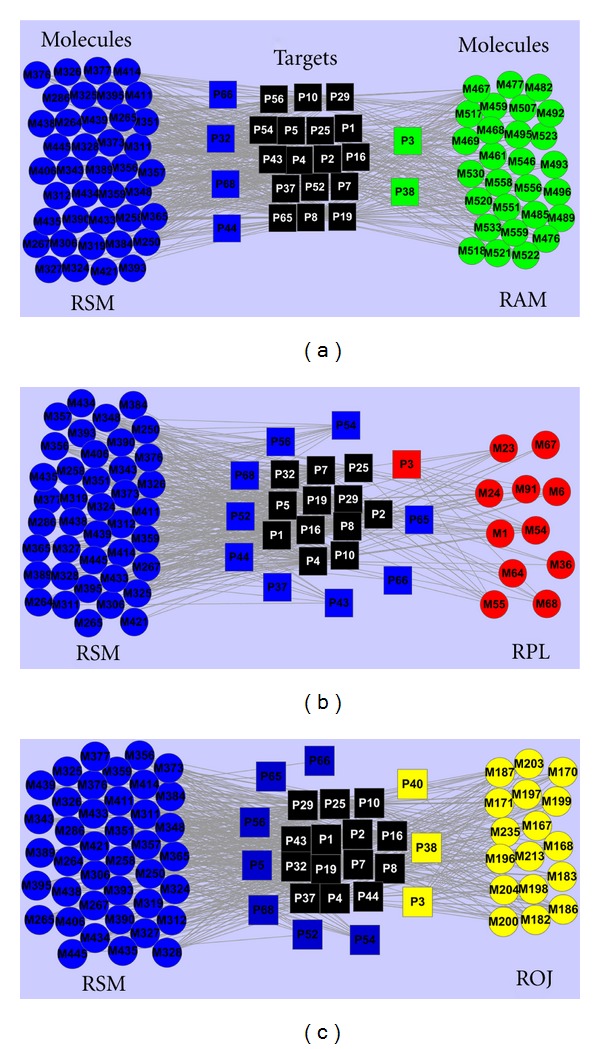
Drug-target interaction networks bioactive molecules from *Radix *Salviae Miltiorrhiza (RSM), Radix Astragali Mongolici (RAM), Radix Puerariae lobatae (RPL), and Radix Ophiopogonis Japonici (ROJ). (a) 40 bioactive compounds (blue circles) from RSM and 28 ones from RAM (green circles) predicted to have 23 potential protein targets (squares). The black squares (17) are the common targets of both herbs. The blue (4) and green (2) squares are the specific targets of RSM and RAM, respectively. (b) 40 bioactive compounds (blue circles) from RSM and 11 ones from RPL (red circles) predicted to have 22 potential protein targets (squares). The black squares (12) are the common targets of both herbs. The blue (9) and red (1) squares are the specific targets of RSM and RAM, respectively. (c) 40 bioactive compounds (blue circles) from RSM and 17 ones from ROJ (yellow circles) predicted to have 24 potential protein targets (squares). The black squares (14) are the common targets of both herbs. The blue (7) and yellow (3) squares are the specific targets of RSM and RAM, respectively.

As mentioned above, a total of 68, 77, 34, and 40 targets were identified for RSM, RAM, RPL, and ROJ, respectively. To elucidate the synergic mechanism of different herbs on treating CVD, all target proteins associated with CVD are used to construct the drug-target network for three herb pairs, that is, RSM and RAM, RSM and RPL, RSM and ROJ, by linking with the cognate compounds (Figures 2(a), 2(b), and 2(c)). For RSM and RAM, Figure 2(a) shows a global view of the bipartite graph with color-coded nodes which correspond to either drugs (circle) or target proteins (square): compounds in RAM (green), compounds in RSM (blue), the same compounds in both herbs (black), targets specific to RAM (green), targets specific to RSM (blue). The network consists of 91 nodes and 529 edges. For compounds, M467 (7-O-methylisomucronulatol) exhibits the highest number of target connections (DD = 17), followed by M258 (2-isopropyl-8-methylphenanthrene-3,4-dione, DD = 15), M348 (dan-shexinkum b, DD = 14), M357 (dihydroisotanshinone*Ⅰ*, DD = 14), M365 (epidanshenspiroketallactone, DD = 14), M459 (3,9-di-O-methylnissolin, DD = 14), while the molecules M384 (manool), M393 (miltirone *Ⅱ*), M477 (sitosterol), M482 (aglycon of *β*-sitosterol-3-O-*β*-D-glucopyranoside daucosterol), M495 (aglycon of alexandrin), M558 (lupenone) have the least targets (DD = 1), The average number of targets per drug is 7.78, indicating the polypharmacology of drugs. For the proteins, the average number of drugs per target is 23. P8 (estrogen receptor) possesses the largest number of connected ingredients (DD = 61), followed by P4 (prostaglandin G/H synthase 2, DD = 60), P7 (nitric oxide synthase, inducible, DD = 52), and P19 (cell division protein kinase 2, DD = 51). Interestingly, 17 targets (73.91%) are found to be shared by both herbs. For examples, the common target P29 (*β*2-adrenergic receptor), which is related to heart failure, hypertension, and ischemic heart disease [69], can be modulated by 21 compounds in RSM and 8 in RAM. Similarly, another important target P2 (prothrombin) that plays an important role in CVD [60] is impacted by 36 chemicals in RSM and 13 in RAM. This suggests that individual drugs in RSM and RAM can act on the same targets in a single formula, thus exert synergistic therapeutic effect on CVD.

CVD is a complex disease in which multiple mediators contribute to overall disease pathogenesis by distinct or redundant mechanisms; drugs designed to act against individual molecular targets probably yield less therapeutic efficacy than simultaneous blockade of multiple targets [35]. Thus another way called multicomponent therapy is also conceivable. Among all 23 targets, we find that RSM possess 4 specific potential targets and RAM has 2 specific potential targets (Figure 2(a)), that is, P66 (alpha-2A adrenergic receptor), P68 (alpha-2B adrenergic receptor), P44 (leukotriene A-4 hydrolase), and P32 (coagulation factor VII), are the specific targets of RAM, while P3 (PPAR*γ*) and P38 (vascular endothelial growth factor receptor 2) are only targeted by RSM. To take target-disease-pathway information into consideration, intriguingly, we find that several targets are involved in the same or related pathway. For example, prostaglandin G/H synthase 1 and prothrombin belong to platelet aggregation inhibitor pathway, which is known to be related to myocardial infarction [70], thus individual drugs in RSM and RAM could act on different targets in the same pathways, thereby having synergistic effect on the treatment of CVD. Furthermore, salvianic acid A in RSM can also target multiple proteins including beta-1/2 adrenergic receptor, nitric-oxide synthase (endothelial), and PPAR*γ* which are responsible for dilating coronary arteries and protecting the myocardium from reperfusion injury of the ischemic heart. This indicates that drugs in both herbs can also act on different targets in related pathways. Combined, these results suggest that both herbs hitting more functionally diverse targets with clinically relevant associations would improve polypharmacology in treating CVD.

For RSM and RPL (Figure 2(b)), a total of 22 protein targets (20 for RSM and 12 for RPL) and 51 molecules (40 for RSM and 11 for RPL) were included in the constructed network, to which 457 reactions (edges) were assigned, that is, 17 drugs, 2 targets, and 72 interactions less than the network constructed from both RSM and RAM. This translates into a significant increase in the number of interactions per drug, increasing the value from 7.78 to 8.96, while for the proteins, the average number of drugs per target decreases from 23.00 to 20.78. This difference may result from the different properties of the chemical space in RAM and RPL which link implicitly to some degree of target promiscuity [71]. Similar to RSM and RAM, 12 targets (54.54%) can be commonly modulated by RSM and RPL. For example, P4 (prostaglandin G/H synthase 2, DD = 48) can be modulated by 39 compounds in RSM and 9 in RPL. Besides, multiple drugs in RSM and RPL can also act on different targets in related pathways in treating CVD. For examples, NO is an important protective molecule in the vasculature, and endothelial nitric-oxide synthase (P5) is responsible for most of the vascular NO [72]. Simultaneously, NO can downregulate cell division protein kinase 2 (P19) activity [73] that has been implicated in prevention of coronary arteriosclerosis [74].

For RSM and ROJ, the drug-target network (Figure 2(c)) was based on the 534 interactions connecting 57 drugs (40 for RSM and 17 for ROJ) to 24 targets (21 for RSM and 17 for ROJ), resulting in an average number of interactions per drug of 9.37. In this network, 14 out of 24 target proteins are connected to both herbs. For example, mitogen-activated protein kinase 14 (P25) targeted by 17 chemicals in RSM and 15 in ROJ plays a protective role against cardiac myocyte apoptosis and myocardial remodeling [75]. These data suggest that both herbs used together to treat CVD can enhance the pharmacological effects by acting at the same molecular target, which is probably more effective than RSM alone.

Taken together, the above results provide insights into that the synergic effect between RSM and other three herbs could result from two strategies: (1) multiple drugs act on the same target; (2) multiple drugs act on different targets in related (or even the same) pathways. Although RSM and other herbs have shown progress as CVD treatments, they probably have distinct mechanistic differences from their different chemical compositions which are the foundation of pharmacology. This suggests that the combination of RSM and the other herbs will show great promise to have synergistic combined effect and overcome drug resistances in CVD therapies. Therefore, different medicinal herbs or drugs of lower potencies need to be appropriately combined in accordance with these profiles and probably in a personalized manner to achieve sufficient levels of efficacy.

## 4. Limitations

In this work, although such approaches have produced a significant amount of knowledge and understanding, only three widely used herb pairs were discussed. As more and more resources have been devoted to uncovering molecular mechanisms of herbal medicine under different conditions, there is a strong need of analysis for more other different herb pairs in a biological meaningful way. Moreover, other factors also have to be considered to evaluate whether two or more herbs can be merged (as a formula): do the herbs act in the same cell type or at the same developmental stage of the cell?

## 5. Conclusion

Herbal medicine has been widely used for disease treatment and is fast becoming a very popular form of alternative medicine worldwide. In TCM, multiple herbs have been frequently used together (multiherb recipe) to execute therapeutic actions. To formulate these TCM recipes, special herb pairs claimed to be unique combinations have been frequently used for achieving synergism that can modulate the efficacy and toxicity of the chemicals of the constituent herbs. However, the mechanisms of synergistic actions of herbal ingredients are still an unresolved issue. Here, we investigate a three representative herb pairs, that is, RSM and RAM, RSM and RPL, RSM and ROJ, by using a novel modeling system that integrates OB and DL screening, targets identification, and network pharmacology. Our results show the following.

1. 43, 44, 12, 19 bioactive ingredients have been identified for the herbs RSM, RAM, RPL, and ROJ, respectively, suggesting that chemical compositions of RSM and the other three herbs have substantially different properties.
2. 21, 19, 13, 17 potential targets associated with CVD have been identified for RSM, RAM, RPL, and ROJ, respectively, which are critical for better understanding of the mechanisms of action of herbs for the treatment of CVD and for the development of novel drugs and TCM modernization.
3. Despite the pharmacological roles of the four herbs being very similar in treatment of CVD, the interaction of individual drugs in each herb with target proteins may be different.
4. In TCM, the synergistic effect between RSM and the other three herbs could result from both strategies including (1) multiple drugs act on the same target; (2) multiple drugs act on different targets in related (or even the same) pathways. Therefore, the mutual enhancement of different herb pairs could be achieved through the different mode of actions to exert a complementary synergistic effect. The discovered mechanisms of synergistic actions of herbal ingredients will offer insights into designing new multi-target drugs and drug combinations and into discovering potent drug combinations that are individually subtherapeutic but efficacious in combination.

Based on the approach developed in this study, we can re-evaluate herbal formulae comprising many species of herbs, thus providing professional advice to support development of recipe optimization of TCM, which will promote drug discovery.

## Supplementary Material

Supplementary Table S1: The information of all 87 protein targets.
Supplementary Figure S2: The profile distributions of eight important molecular properties for all molecules from RSM, RAM, RPL and ROJ.

## Figures and Tables

**Table 1:** 118 bioactive compounds from four herbs RSM, RAM, RPL, and ROJ and corresponding predicted oral bioavailability (OB), and drug-likeness (DL).

NO.	Compound	DL	OB	Herbs*
M161	ophiogenin	0.76	100.00	ROJ
M306	przewalskin b	0.44	100.00	RSM
M476	dimethyl-4,4′-dimethoxy-5,6,5′,6′-mdimethylene-dioxybiphenyl-2,2′-dicarboxylate	0.67	100.00	RAM
M465	7,2′-dihydroxy-3′,4′-dimethoxyisoflavone-7-O-*β*-D-glucoside	0.86	99.41	RAM
M6	tuberosin	0.76	95.05	RPL
M196	5-hydroxy-7,8-dimethoxy-6-methyl-3-(3′,4′-dihydroxybenzyl)chroman-4-one	0.41	93.42	ROJ
M521	Calycosin	0.24	88.9	RAM
M267	4-hydroxy-1-vinylcarboxy-7-(3,4-dihydroxyphenyl)benzo-*β*-furan	0.31	88.57	RSM
M462	4′-O-beta-Glucopyranosyl-5-O-Methylvisamminol	0.81	83.95	RAM
M204	N-[*β*-hydroxy-*β*-(4-hydroxy)phenyl]ethyl-4-hydroxy cinnamide	0.23	82.91	ROJ
M468	9,10-dimethoxypterocarpan-3-O-*β*-D-glucoside	0.92	81.91	RAM
M411	przewalskinone b	0.27	81.61	RSM
M182	ophiopogonanone F	0.45	81.49	ROJ
M505	astragaloside IV	0.15	81.32	RAM
M493	riboflavin	0.5	81	RAM
M480	betulinic acid	0.78	78.63	RAM
M510	aglycon of astragaloside I	0.2	77.02	RAM
M433	tanshindiol a	0.46	75.49	RSM
M183	ophiopogonanone G	0.46	75.41	ROJ
M533	aglycon of rhamnocitrin-3-O-glucoside	0.27	75.39	RAM
M461	5,4′-dihydroxy-3,7-dimethoxyflavone kumatakenin	0.29	75.36	RAM
M550	isoquercitrin	0.77	75.03	RAM
M509	astragaloside I	0.11	73.23	RAM
M55	8-prenylgenistein	0.37	72.6	RPL
M286	formyltanshinone	0.42	72.34	RSM
M390	miltionone II	0.44	71.03	RSM
M467	7-O-methylisomucronulatol	0.3	70.95	RAM
M464	aglycon of 5′-hydroxyiso-muronulatol-2′,5′-di-O-glucoside	0.8	70.73	RAM
M365	epiRSMspiroketallactone	0.31	68.27	RSM
M187	ophiopogonone B	0.31	67.52	ROJ
M559	red sandalwood ene	0.48	66.61	RAM
M492	behenic acid	0.26	65.99	RAM
M530	kaempferol	0.24	65.98	RAM
M507	mucronulatol-7-O-glucoside	0.86	65.21	RAM
M328	tanshinol I	0.52	64.81	RSM
M200	6-aldehydoisoophiopogonone B	0.38	64.39	ROJ
M406	prolithospermic acid	0.31	64.3	RSM
M523	aglycon of calycosin-7-O-glucoside	0.24	64.29	RAM
M501	astragaloside III	0.1	63.07	RAM
M485	aglycon of formononetin-7-glucoside	0.21	62.54	RAM
M478	*γ*-aminobutyric acid	0.01	62.12	RAM
M439	tanshinone VI	0.3	61.7	RSM
M213	ophiopogonanone B	0.3	59.58	ROJ
M421	salvianolic acid g	0.61	59.36	RSM
M522	calycosin-7-O-glucoside	0.81	58.36	RAM
M327	tanshinol II	0.56	58.29	RSM
M198	6-aldehydoisoophiopogonanone B	0.38	58.26	ROJ
M459	3,9-di-O-methylnissolin	0.48	57.75	RAM
M512	aglycon of astragaloside II	0.25	56.75	RAM
M516	rutin	0.68	56.65	RAM
M376	isotanshinone IIb	0.45	56.64	RSM
M551	aglycon of isoquercitrin	0.28	56.54	RAM
M54	8-prenyldaidzein	0.33	56	RPL
M351	danshenspiroketallactone	0.31	55.99	RSM
M311	przewaquinone c	0.4	55.83	RSM
M373	isocryptotanshinone	0.39	55.08	RSM
M197	6-aldehydo-7-methoxyl-isoophiopogonanone B	0.41	54.45	ROJ
M326	tanshinol a	0.41	54.27	RSM
M546	folic acid	0.71	53.33	RAM
M325	tanshinaldehyde	0.45	52.54	RSM
M395	neocryptotanshinone	0.32	52.54	RSM
M343	cryptotanshinone	0.4	52.44	RSM
M495	aglycon of alexandrin	0.75	52.12	RAM
M556	biochain B	0.21	51.72	RAM
M482	aglycon of *β*-sitosterol-3-O-*β*-D-glucopyranoside daucosterol	0.75	50.29	RAM
M377	isotanshinone IIa	0.4	50.02	RSM
M389	miltionone I	0.32	49.68	RSM
M506	aglycon of astragaloside IV	0.32	49.67	RAM
M356	deoxyneocryptotanshinone	0.29	49.51	RSM
M554	isomucronulatol-7,2′-di-O-glucosiole	0.62	49.32	RAM
M324	aglycon of tannin	0.26	49.23	RSM
M1	(Z,Z,Z)-8,11,14-eicosatrienoic acid	0.2	48.76	RPL
M469	aglycon of 9,10-dimethoxypterocarpan-3-O-*β*-D- glucoside	0.42	47.86	RAM
M96	daidzein-4′,7-diglucoside	0.67	47.27	RPL
M348	danshexinkum b	0.26	46.79	RSM
M270	6-O-syringyl-8-O-acetyl shanzhiside methyl ester	0.71	46.69	RSM
M532	rhamnocitrin-3-O-glucoside	0.76	45.82	RAM
M36	3′-methoxydaidzin	0.81	45.13	RPL
M264	3*α*-hydroxytanshinone IIa	0.44	45.1	RSM
M384	manool	0.2	45.06	RSM
M359	dihydrotanshinone I	0.36	45.04	RSM
M393	miltirone II	0.24	44.95	RSM
M477	sitosterol	0.78	44.72	RAM
M430	stigmasterol	0.76	43.83	RSM
M319	sclareol	0.21	43.67	RSM
M445	Δ1-dehydrotanshinone	0.4	43.67	RSM
M518	chlorogenic acid	0.33	43.43	RAM
M357	dihydroisotanshinone I	0.36	43.39	RSM
M435	tanshindiol c	0.45	42.87	RSM
M434	tanshindiol b	0.45	42.68	RSM
M558	lupenone	0.78	42.39	RAM
M265	3*β*-hydroxytanshinone IIa	0.45	42.17	RSM
M312	przewaquinone d	0.45	41.31	RSM
M168	5,7-dihydroxy-6,8-dimethyl-3-(2′-hydroxy-3′,4′-methylenedioxybenzyl)chromone	0.53	41.14	ROJ
M258	2-isopropyl-8-methylphenanthrene-3,4-dione	0.23	41.06	RSM
M511	astragaloside II	0.13	40.87	RAM
M489	syringaresinol	0.72	40.79	RAM
M91	soyasapogenol C	0.77	40.74	RPL
M167	5,7,2′-trihydroxy-8-methyl-3-(3′,4′-methylenedioxyb-enzyl)chromone	0.49	40.63	ROJ
M171	methylophiopogonone B	0.34	40.52	ROJ
M250	1,2,5,6-tetrahydrotanshinone	0.36	40.5	RSM
M517	lariciresinol	0.38	40.27	RAM
M170	methylophiopogonone A	0.48	40.24	ROJ
M520	aglycon of ononin	0.21	38.22	RAM
M23	daidzein	0.19	38.19	RPL
M414	salvianic acid a	0.06	35.95	RSM
M24	formononetin	0.21	32.3	RPL
M438	tanshinone IIb	0.45	21.7	RSM
M119	ophiopogonin D	0.06	20.86	ROJ
M203	methylophiopogonanone A	0.48	19.1	ROJ
M186	ophiopogonone A	0.44	14.24	ROJ
M496	quercetin	0.28	13.18	RAM
M64	puerarin	0.69	12.92	RPL
M199	6-aldehydoisoophiopogonone A	0.53	12.6	ROJ
M67	daidzin	0.73	9.83	RPL
M68	formononetin-7-O-b-D-glycoside-ononin	0.78	8.62	RPL
M323	tannin	0.03	7.3	RSM
M235	methylophiopogonanone B	0.34	5.26	ROJ

*RSM (Radix Salviae Miltiorrhiza), RAM (Radix Astragali Mongolici), RPL (Radix Puerariae lobatae), and ROJ (Radix Ophiopogonis Japonici).

**Table 2:** Comparison of molecular properties between RSM, RAM, RPL, and ROJ.

Index	RSM (mean ± SD)		RAM (mean ± SD)		ROJ (mean ± SD)		RPL (mean ± SD)	
	Total compounds	Active compounds	Total compounds	Active compounds	Total compounds	Active compounds	Total compounds	Active compounds
nHDon	2.09 (2.69)	1.95 (3.88)	4.05 (2.86)	3.89 (2.77)	3.22 (3.23)	2.68 (1.49)	5.00 (4.65)	3.42 (2.19)
nHAcc	4.23 (4.47)	5.19 (6.78)	7.51 (4.70)	7.91 (4.32)	6.72 (6.22)	6.58 (2.46)	8.50 (7.62)	6.42 (3.70)
MLogP	2.45 (2.61)	2.10 (2.28)	0.75 (2.59)	1.18 (2.60)	2.12 (2.48)	1.61 (0.91)	1.28 (3.38)	1.44 (2.57)
MW	310.37 (156.93)	343.28 (219.76)	411.00 (232.39)	438.31 (169.88)	428.72 (262.11)	373.42 (120.83)	484.18 (286.37)	379.77 (92.05)
RBN	3.66 (5.17)	2.09 (4.99)	5.17 (3.68)	5.02 (3.42)	4.99 (4.81)	3.26 (1.45)	6.58 (6.02)	4.08 (3.96)
nCIC	2.74 (1.73)	3.72 (1.35)	3.59 (2.66)	4.14 (1.84)	4.05 (3.01)	3.74 (1.52)	3.89 (2.6971)	3.58 (1.38)
Hy	0.56 (2.00)	0.38 (2.79)	1.92 (2.06)	1.68 (1.95)	1.13 (2.13)	0.74 (0.97)	2.46 (3.28)	1.28 (1.61)
TPSA (Tot)	73.61 (76.27)	86.86 (114.01)	120.61 (72.73)	123.18 (69.35)	102.76 (92.12)	100.13 (35.49)	142.01 (125.68)	106.28 (59.74)

*RSM (Radix Salviae Miltiorrhiza), RAM (Radix Astragali Mongolici), RPL (Radix Puerariae lobatae), and ROJ (Radix Ophiopogonis Japonici).
