# Supplementary material for: A Systems Biology Approach to Uncovering Pharmacological Synergy in Herbal Medicines with Applications to Cardiovascular Disease
Source: Evid Based Complement Alternat Med. 2012 Nov 29;2012:519031. doi: 10.1155/2012/519031 (PMC3518963; doi:10.1155/2012/519031)
Supplement: Supplementary file 1 — Supplementary Table S1: The information of all 87 protein targets. Supplementary Figure S2: The profile distributions of eight important molecular properties for all molecules from RSM, RAM, RPL and ROJ. [file 519031.f1.doc]

**Table S1. The information of all 87 protein targets**

| NO. | target |
| --- | --- |
| P1* | Prostaglandin G/H synthase 1 |
| P2* | Prothrombin |
| P3* | Peroxisome proliferator-activated receptor gamma |
| P4* | Prostaglandin G/H synthase 2 |
| P5* | Nitric-oxide synthase, endothelial |
| P6 | Dipeptidyl peptidase 4 |
| P7* | Nitric oxide synthase, inducible |
| P8* | Estrogen receptor |
| P9 | Androgen receptor |
| P10* | Sodium channel protein type 5 subunit alpha |
| P11 | Coagulation factor X |
| P12 | Carbonic anhydrase 2 |
| P13 | Calmodulin |
| P14 | Acetylcholinesterase |
| P15 | DNA topoisomerase 2-alpha |
| P16* | Estrogen receptor beta |
| P17 | Glycogen synthase kinase-3 beta |
| P18 | Heat shock protein HSP 90-alpha |
| P19* | Cell division protein kinase 2 |
| P20 | Proto-oncogene serine/threonine-protein kinase Pim-1 |
| P21 | Trypsin-1 |
| P22 | Gag-Pol polyprotein |
| P23 | Cyclin-A2 |
| P24 | Retinoic acid receptor RXR-alpha |
| P25* | Mitogen-activated protein kinase 14 |
| P26 | Serine/threonine-protein kinase Chk1 |
| P27 | cAMP-dependent protein kinase catalytic subunit alpha |
| P28 | Tyrosine-protein phosphatase non-receptor type 1 |
| P29* | Beta-2 adrenergic receptor |
| P30 | Sodium-dependent serotonin transporter |
| P31 | cAMP-dependent protein kinase inhibitor alpha |
| P32* | Coagulation factor VII |
| P33 | Nuclear receptor coactivator 2 |
| P34 | Glucocorticoid receptor |
| P35 | Mineralocorticoid receptor |
| P36 | Alpha-1B adrenergic receptor |
| P37* | Potassium voltage-gated channel subfamily H member 2 |
| P38* | Vascular endothelial growth factor receptor 2 |
| P39 | Calcium-activated potassium channel subunit alpha 1 |
| P40* | Peroxisome proliferator-activated receptor delta |
| P41 | Ig gamma-1 chain C region |
| P42 | Nuclear receptor coactivator 1 |
| P43* | Phosphatidylinositol-4,5-bisphosphate 3-kinase catalytic subunit gamma isoform |
| P44* | Leukotriene A-4 hydrolase |
| P45 | D(1A) dopamine receptor |
| P46 | Muscarinic acetylcholine receptor M3 |
| P47 | Muscarinic acetylcholine receptor M1 |
| P48 | Muscarinic acetylcholine receptor M5 |
| P49 | 5-hydroxytryptamine 3 receptor |
| P50 | Muscarinic acetylcholine receptor M4 |
| P51 | Delta-type opioid receptor |
| P52* | 5-hydroxytryptamine 2A receptor |
| P53 | Alpha-1A adrenergic receptor |
| P54* | Muscarinic acetylcholine receptor M2 |
| P55 | Sodium-dependent dopamine transporter |
| P56* | Alpha-1D adrenergic receptor |
| P57 | Mu-type opioid receptor |
| P58 | Gamma-aminobutyric-acid receptor subunit alpha-1 |
| P59 | Neuronal acetylcholine receptor subunit alpha-7 |
| P60 | Progesterone receptor |
| P61 | Neuronal acetylcholine receptor subunit alpha-2 |
| P62 | cGMP-inhibited 3',5'-cyclic phosphodiesterase A |
| P63 | D(2) dopamine receptor |
| P64 | Beta-lactamase |
| P65* | Beta-1 adrenergic receptor |
| P66* | Alpha-2A adrenergic receptor |
| P67 | Alpha-2C adrenergic receptor |
| P68* | Alpha-2B adrenergic receptor |
| P69 | Amine oxidase [flavin-containing] B |
| P70 | Chymotrypsinogen B |
| P71 | Retinoic acid receptor RXR-beta |
| P72 | Hepatocyte growth factor receptor |
| P73 | Delta-aminolevulinic acid dehydratase |
| P74 | Alcohol dehydrogenase 1A |
| P75 | 4-aminobutyrate aminotransferase, mitochondrial |
| P76 | Glycine amidinotransferase, mitochondrial |
| P77 | Alcohol dehydrogenase 1B |
| P78 | Alcohol dehydrogenase 1C |
| P79 | Cathepsin D |
| P80 | Aspartate aminotransferase |
| P81 | Haloalkane dehalogenase |
| P82 | Cytochrome P450-cam |
| P83 | Ferrichrome-iron receptor |
| P84 | Bacillolysin |
| P85 | Trypsin-3 |
| P86 | Cholinesterase |
| P87 | 2-hydroxy-6-oxo-7-methylocta-2,4-dienoate hydrolase |

*represent the target associated with CVD


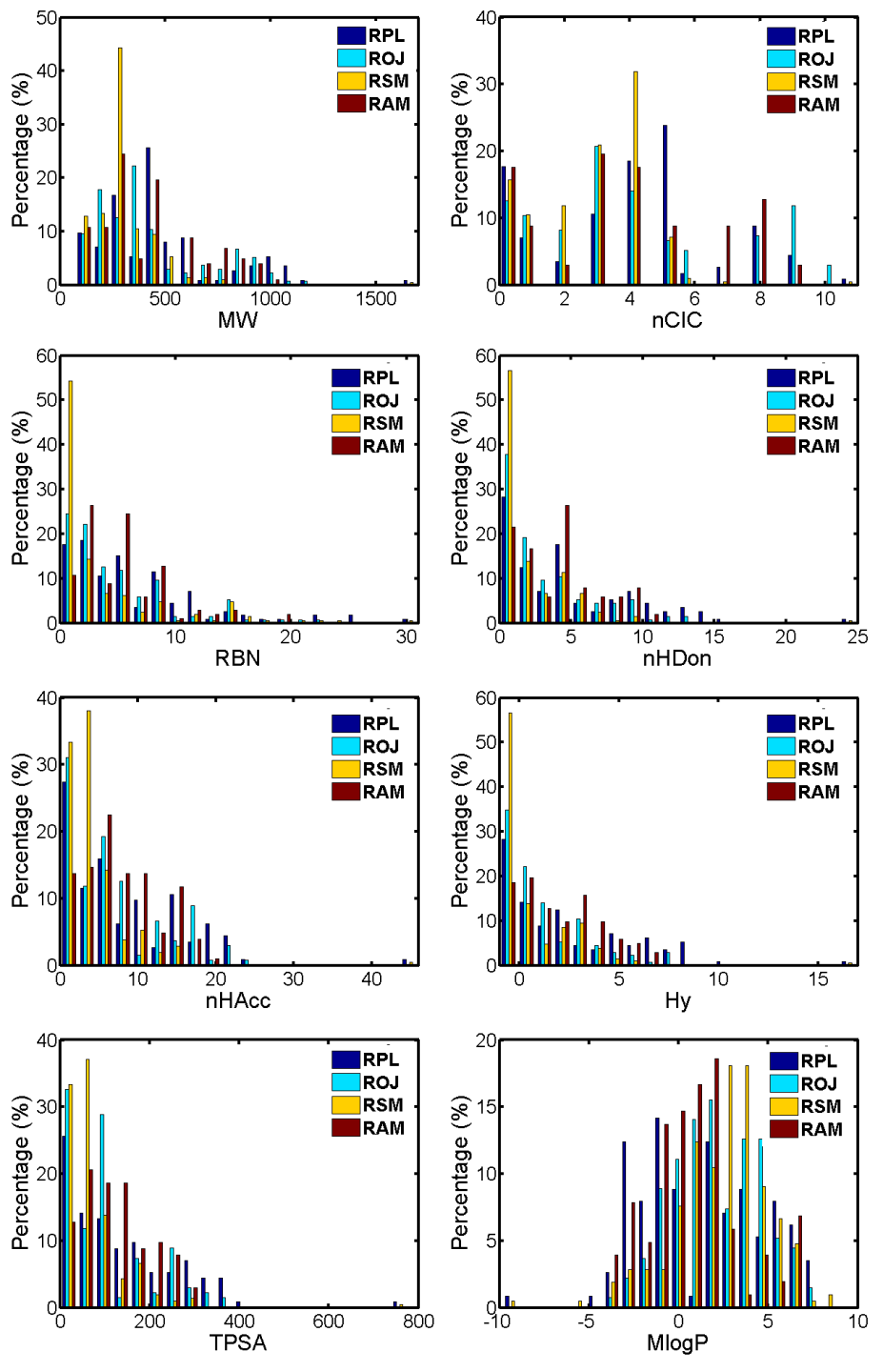


**Figure S1. The profile distributions of eight important molecular properties for all molecules from RSM, RAM, RPL and ROJ.**
